# Injury Biomechanics Evaluation of a Driver with Disabilities during a Road Accident—A Numerical Approach

**DOI:** 10.3390/ma15227956

**Published:** 2022-11-10

**Authors:** Kamil Sybilski, Fábio A. O. Fernandes, Mariusz Ptak, Ricardo J. Alves de Sousa

**Affiliations:** 1 Institute of Mechanics and Computational Engineering, Faculty of Mechanical Engineering, Military University of Technology, gen. Sylwestra Kaliskiego 2, 00-908 Warsaw, Poland; 2 TEMA—Centre for Mechanical Technology and Automation, Department of Mechanical Engineering, University of Aveiro, Campus de Santiago, 3810-193 Aveiro, Portugal; 3 LASI—Intelligent Systems Associate Laboratory, Portugal; 4 Faculty of Mechanical Engineering, Wroclaw University of Science and Technology, Lukasiewicza 7/9, 50-371 Wroclaw, Poland

**Keywords:** biomechanics, head, brain injury, vehicle safety, road accident, finite element analysis

## Abstract

Numerical methods are often a robust way to predict how external mechanical loads affect individual biological structures. Computational models of biological systems have been developed over the years, reaching high levels of detail, complexity, and precision. In this study, two cases were analysed, differing in the airbag operation; in the first, the airbag was normally activated, and in the second case, the airbag was disabled. We analysed a model of a disabled person without a left leg who steers a vehicle using a specialized knob on the steering wheel. In both cases, a head-on collision between a car moving at an initial speed of 50 km/h and a rigid obstacle was analysed. We concluded that the activated airbag for a person with disabilities reduces the effects of asymmetries in the positioning of the belts and body support points. Moreover, all the biomechanical parameters, analysed on the 50th percentile dummy, i.e., HIC, seat belt contact force and neck injury criterion (Nij) support the use of an airbag. The resulting accelerations, measured in the head of the dummy, were induced into a finite element head model (YEAHM) to kinematically drive the head and simulate both accidents, with and without the airbag. In the latter, the subsequent head injury prediction revealed a form of contrecoup injury, more specifically cerebral contusion based on the intracranial pressure levels that were achieved. Therefore, based on the in-depth investigation, a frontal airbag can significantly lower the possibility of injuries for disabled drivers, including cerebral contusions.

## 1. Introduction

Nowadays, 15% of the world’s population lives with some form of disability [1]. Between 110–190 million adults have serious functional problems. The number of people with disabilities continues to rise, which is related to increased life expectancy and the number of accidents, including serious traffic accidents [2]. Acquiring a disability is associated with reduced independence and ability to work and perform social functions [2].

In recent years, much attention has been paid to the activation of people with disabilities. Many works have been written on various aspects of the lives of people with disabilities spending leisure time [3], and adjusting infrastructure [4,5,6]. Thanks to, among other things, national programs, and the concept of universal design, it has been possible to adapt many environments to the widest possible group of people, thus increasing equality of opportunity and activation of people with disabilities. Being able to get to work, social gatherings or simple daily shopping on one’s own is a key aspect for everyone, especially for people with disabilities [7,8]. In this regard, the situation is superlative in large cities, where, in addition, there is modern public transportation. In smaller cities and villages, individual transportation plays a significant role.

Cars are not adapted to the needs of people with disabilities. Additional adaptations are needed that change the ergonomics and biomechanics of the driver. Adaptations in cars do not have to complete advanced testing, so there is no confirmation of their safety of use. Hence, it is necessary to conduct research to verify the safety of people with disabilities.

The development of numerical methods makes it possible to conduct reliable simulations of dangerous situations on the road [9]. In the literature can be found many works on this topic. Some researchers focus on developing numerical models of vehicles [10], and others on road infrastructure [11]. Considerable support is provided by commercial dummy models, which undergo many validation tests [12,13,14].

The use of accurate dummy models requires very high computing power and specialised workstations for pre- and post-processing. Therefore, a common solution is to simulate a large phenomenon with a simplified model and then transfer the loads to an accurate model of selected components. In biomechanical studies, this allows the accurate determination of injuries to internal organs. Examples of such work include [15,16,17,18,19]. Such an approach was used in this article.

## 2. Materials and Methods

### 2.1. Global Analyses

**Figure 1:**
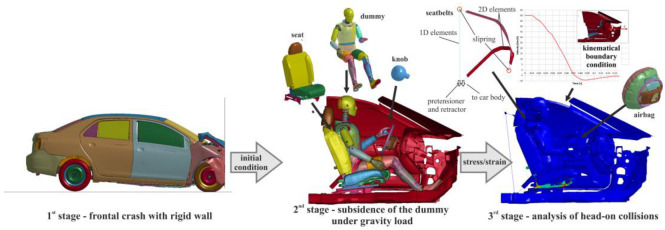
Numerical research strategy [21] (with permission of Editor in Chief of Acta of Bioengineering and Biomechanics).

In the article, the numerical study was divided into two parts. In the first, the accelerations occurring in the dummy’s head during a head-on collision were determined, while in the second, the determined accelerations were used to accurately analyse brain injuries. An important aspect accompanying the conducted research was the faithful reproduction of real conditions. Therefore, each of the analyses carried out in the first stage was divided into three stages: a frontal collision of the entire validated vehicle [20] with a rigid obstacle, subsidence of the validated 50 percentile dummy (Humanetics Hybrid III 50th male) [13] on the seat and instrumentation, and final analysis of the dummy’s behaviour during the collision (Figure 1). All numerical analyses in the first stage were carried out using an explicit time integration step in the LS-Dyna solver (ANSYS, Livemore, CA, USA). In order to significantly reduce computation time, in the first stage the entire vehicle was analysed without the dummy, while in the second and third stages the analyses were carried out for a slice of the vehicle. This approach was used due to the lack of significant deformation of the vehicle’s interior during a frontal collision with an initial speed of 50 km/h [21]. The first stage was designed to provide deceleration curves for the third stage.

**Figure 2:**
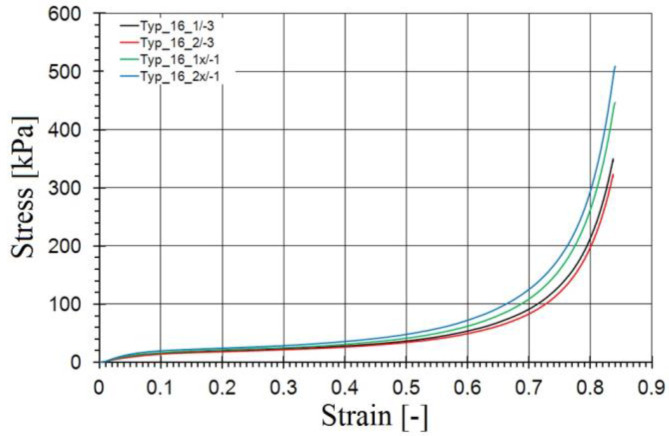
Stress vs. volumetric strain curves for seat foams, based on [23].

In the second stage, the full subsidence of the dummy on the additional equipment and vehicle interior components under the action of gravity and external forces was realised. The car body was modelled as a rigid body. All other elements were modelled as deformable. The highest deformation in this stage was in the seat foam. A proper description of the seat cushions required experimental tests to determine material parameters for the constitutive model. The experimental tests were carried out in accordance with the regulations included in ASTM C165-00 (standard test method for measuring compressive properties of thermal insulations) [22]. Based on them, cuboid samples (100.0 mm × 100.0 mm × 50.0 mm) were prepared [23]. Then, the samples were uniaxially compressed on Instron 8802 testing machine (Norwood, MA, USA). The results of the performed tests were stress vs. strain curves shown in Figure 2.

The dummy at the analysis preparation stage was placed in a near-target position with a few millimetres of clearance so that it did not come into contact with the vehicle components. Then, the gravitational acceleration acting vertically on all components in the numerical model and the accelerations acting on selected parts of the dummy were defined. Additional accelerations were designed to slide the left hand over the knob on the steering wheel. A contact taking into account frictional forces was determined between the dummy and all components of the car. Settlement analyses were conducted until the dummy’s position was fully stabilised and all oscillations of the dummy’s centre of gravity position stopped. During this process, the scaling curve of the value of additional accelerations gradually decreased its value from 1 to 0. In the final stage of subsidence, the upper limb was held in its target positions only as a result of frictional forces. Stage two of the analysis allowed the deformation of the seat and the interaction and frictional forces at the dummy–vehicle interface to be transferred to the third stage (Figure 1).

**Figure 3:**
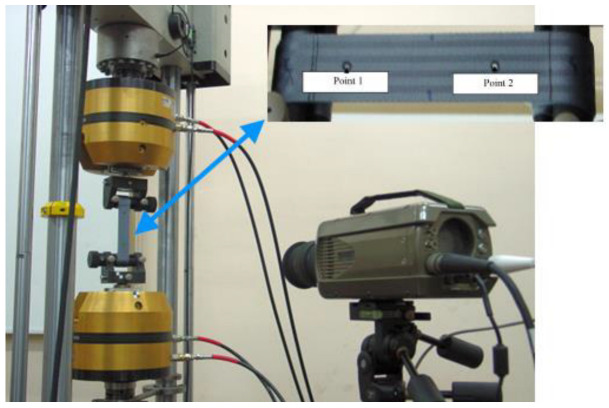
Stress vs. volumetric strain curves for seat foams, based on [21] (with permission of Editor in Chief of Acta of Bioengineering and Biomechanics).

In the third stage of analysis, the initial boundary conditions were changed in the model from stage two, and safety system components were added. The model was completed with a full model of the seat belts, airbags and acceleration sensors. The seat belts were modelled as a combination of 2D and 1D elements. Shell elements were used where the belts came into contact with the dummy’s body. On the other hand, 1D elements were used where connections, buckles, retractors and tensioners occur. This made it possible to obtain the effect of the belts scrolling through the fasteners (when one belt shortened, the other lengthened) [24] and the retraction of the belts through the retractor and tensioner. The full performance characteristics of the pyrotechnic tensioner were used in the numerical model. In order to properly describe the behaviour of seat belts, an experimental study of a quasi-static tensile test was conducted. The tests were carried out using a high-speed Phantom V12 camera and a video-extensometer recording the displacement of two markers (Figure 3). The tape was mounted on the testing machine using handles with special capstans around which the tape was wrapped (Figure 3) [21,25].

**Figure 4:**
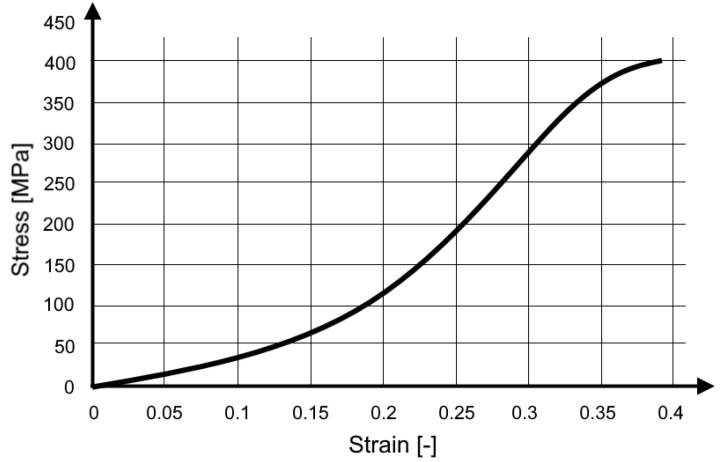
Stress vs. strain curve for the seat belt material [21] (with permission of Editor in Chief of Acta of Bioengineering and Biomechanics).

After the test was completed, the results were analysed using the DIC (digital image correlation) method, which made it possible to determine the longitudinal strain of the tape (Figure 4).

During the third stage, the retractor maintained seat belt tension during the initial phase of the analysis. When the predetermined level of deceleration was exceeded, the acceleration sensor activated the operation of the pretensioner, which shortened the belt and significantly increased its tension to a predetermined value. The occurrence of increased belt tension resulted in the gradual release of the seat belt.

During the numerical analyses, two cases were analysed, differing in the operation of the airbag, in the first, the airbag was normally activated and in the second, it was disabled. In both cases, we analysed a disabled person without a left leg (a medium-sized stump, removed 60% of the length of the thigh) using a knob on the steering wheel. In the dummy model, amputation was achieved by removing the corresponding finite elements.

### 2.2. Head Numerical Model

In order to carry out the detailed analysis of the brain injury, based on the boundary conditions obtained from the global crash test simulation, a validated finite element head model was used, the YEAHM model. The version of YEAHM employed in this work is the original one presented in [26] since the objective is not to predict skull fractures or rupture of cerebral vasculature [27,28], but rather focus on diffuse brain injuries. Nevertheless, the original version is validated against Nahum et al. [29] and Hardy et al. [30] post-mortem human subjects (PMHS) [26,31].

This version is mainly composed of a linear elastic skull, visco-hyperelastic brain and hyperelastic cerebral spinal fluid (CSF). Nevertheless, the skull bone, in this case, is remodelled to be rigid, as in the simulation of Hardy’s experiments [31]. This is justified as unlike in the dummy approach, during the head numerical model simulation, there is no direct contact or impact on the skull. In other words, the accelerations obtained from the crash test simulations were induced on the skull as kinematic boundary conditions.

Regarding the modelling of brain tissue and CSF, nonlinearities were considered. In the case of the brain matter, the characteristic time scale was considered, based on strain rates in the range of 10–100 s^−1^ and compressive strain levels of 10–50%, typical values of brain injuries in impact trauma [32,33,34,35]. These are based on the characterisation performed by Rashid et al. [36,37]. Although the brain tissue samples tested by Rashid et al. [36,37] were from porcine, Nicolle et al. [38] and Thibault and Margulies [39] observed no significant difference between the mechanical properties of human and porcine brain matter. In a recent study, MacManus et al. [40] suggested the use of pig or mouse brain tissue as suitable surrogates to characterise human brain tissue after performing indentation force-relaxation experiments on mouse, rat, pig, and human brains at 10/s strain rate up to 35% strain to determine the dynamic mechanical properties of brain tissue.

Rashid et al. [36,37] determined the mechanical properties of fresh brain tissue by performing unconfined compression tests and tensile tests at strain rates up to 90 s^−1^ and strains up to 30% and also relaxation tests to determine the time-dependent material parameters. Rashid et al. [36,37] estimated optimal parameters for one-term Ogden hyperelastic model and for Prony series, which provided an excellent fitting to the experimental data. The parameters used in this research are based on the ones determined by Rashid et al. [36,37].

CSF is modelled as a solid with very low shear modulus by employing the Mooney–Rivlin strain energy potential. In this study, the relation of C_10_ = 0.9 × C_01_ was used based on the literature [41].

In summary, brain tissue is a very soft, incompressible, strain rate sensitive, nonlinear viscoelastic material. Therefore, a hyperelastic model was used to describe the nonlinear elasticity, combined with a viscoelastic model to describe the time-dependent behaviour, based on characterisation performed by Rashid et al. [36,37]. More details regarding mesh, model of materials, full material data, contact properties and formulation can be found in [26,31].

## 3. Results and Discussion

### 3.1. Global Analyses

The results of the full frontal crash performed in the first stage and the subsidence are presented in [21]. In the third stage, the initial course of both cases is very similar. The body of the vehicle begins to decelerate rapidly, and the body of the dummy moves on the seat towards the steering wheel increasing the pressure on the seat belt. The dummy’s knee hits the elements of the dashboard, the right hand is thrown forward under the influence of the acting deceleration, and the left hand clasps the handle on the steering wheel. In further analysis, in the case without an airbag, the left hand increases pressure on the handle and the head strikes the steering wheel rim, after which the direction of movement of the dummy relative to the seat is changed and movement toward the back begins. In the case of an airbag, the inflating cushion pushes the dummy’s left hand toward the window. The dummy’s body, including the head, hits the inflated cushion and, after completely losing velocity, changes the direction of motion and begins moving toward the seat back.

**Figure 5:**
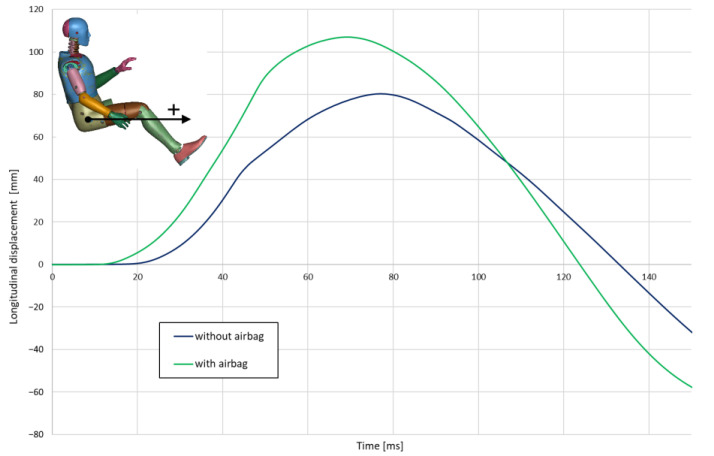
Longitudinal displacement of H-point.

The effects described above are reflected in the longitudinal displacement of the dummy (Figure 5). In the case of the airbag in operation, these displacements are about 33% greater.

**Figure 6:**
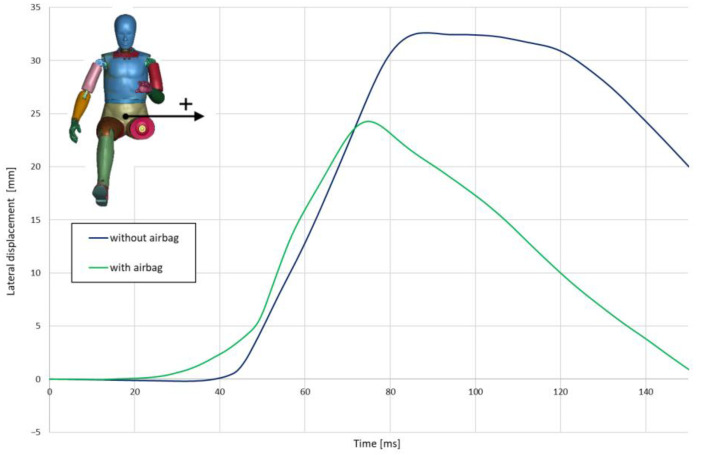
Lateral displacement of H-point.

During a crash in the absence of an airbag, the left hand is blocked continuously against the knob, causing the driver’s torso to rotate and move significantly toward the door (Figure 6). When the airbag is operating in the initial stage, the left hand is pushed out of the steering wheel space and the torso pushes against the airbag (the hands do not provide significant resistance). On this basis, it can be concluded that the airbag reduces the effect of asymmetry in the positioning of the belts and body support points (left hand on the knob, right leg resting on the floor).

**Figure 7:**
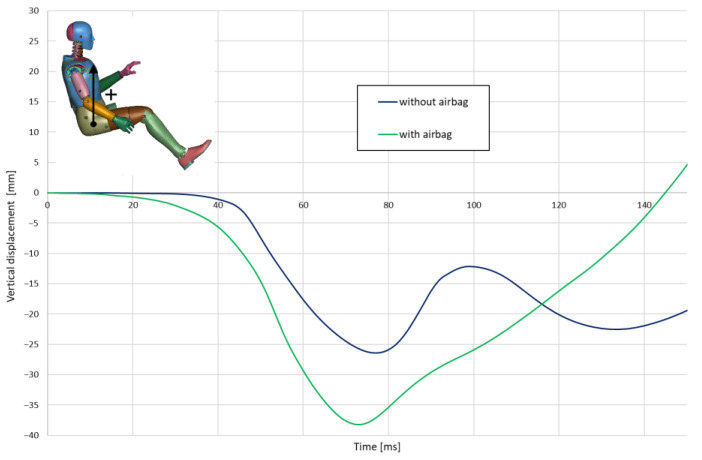
Vertical displacement of H-point.

The action of the airbag and the difference in the left hand change in position also significantly affect the vertical displacement of the driver’s body centre. During a crash, when the airbag is operating, his body is pressed harder into the seat (Figure 7).

**Figure 8:**
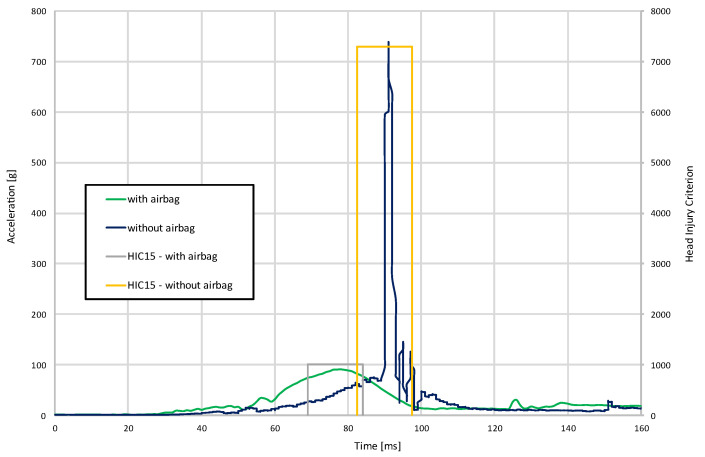
Resultant acceleration and head injury criterion.

In crashes with the airbag in operation, significantly lower head accelerations were recorded, which translates into lower values for the head injury criterion (Figure 8). Without the airbag, the head injury criterion (HIC15) exceeded 7000. When the post-airbag is activated, it decreases to about 1000.

**Figure 9:**
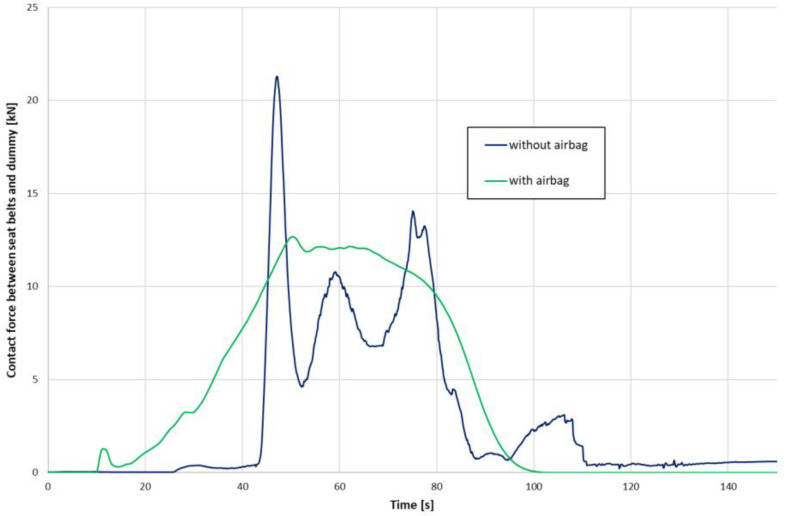
Contact force between seat belts and dummy.

The contact force between the seat belts and the dummy turned out to be smoother for the case with the activated airbag (Figure 9). Moreover, the registered maximum value for the airbag case was approx. 40% lower than the forces acting on the dummy without the airbag in operation.

**Figure 10:**
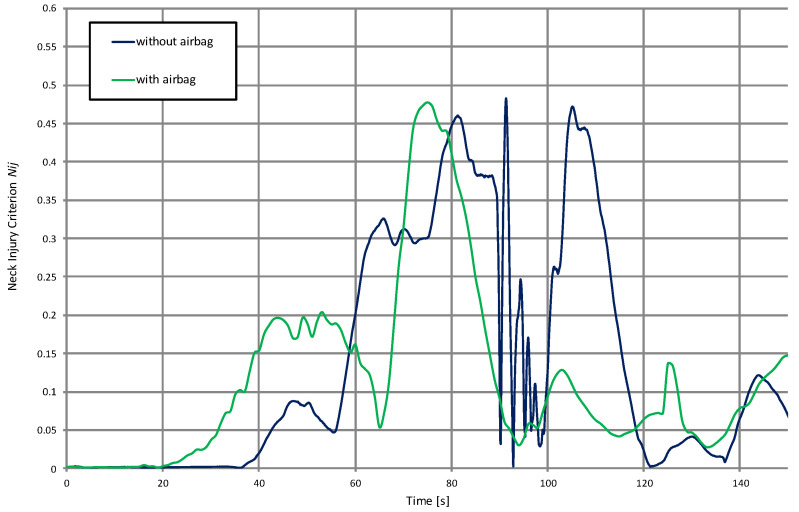
Neck injury criterion (Nij) for the case with and without the activated airbag.

Since the severity of cervical spine injuries is also important, the neck injury criterion (Nij) was also registered to provide a quantitative relationship between forces and moments of the upper neck with accompanied injury risk (Figure 10). Even though the maximum values for both cases stay at the same level, the airbag case features only one peak which is recognised as a safer situation than repeated neck tension/compression. In the case without an airbag, we have three peaks. The first and last are related to the bending of the neck due to the impact and bouncing of the head on the steering wheel. The middle peak is the result of strong lateral displacement of the whole body, which also results in lateral displacement of the head. This, in turn, results in the rotation of the head at the moment of impact with the steering wheels and a strong short-term torsion of the neck.

### 3.2. Brain Injury Analyses

**Figure 11:**
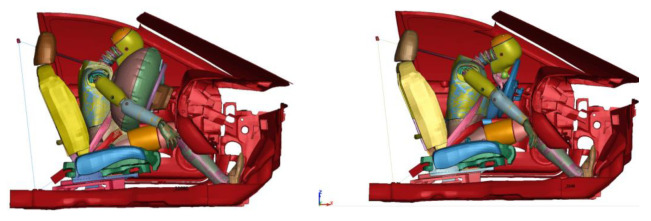
The investigated head injury cases for the impacts: head–airbag at 389 ms (**left**) and the head–steering wheel at 396 ms (**right**) after the start of the simulations.

The output data for the dummy’s CoG of the head was registered as six different acceleration functions in time (three translational and three rotational) and was further used as input to the centre of mass in the finite element head model (FEHM). The skull, in this approach, was modelled as a rigid body as it is widely justified that the skull’s strain is negligible compared to the brain tissues [17,42]. As it was completed for the full dummy model, we present the results for two cases: (1) with the deployed airbag and (2) without the activated airbag. The figure depicted below presents the state at which the registered absolute hydrostatic pressure (MPa) in the brain (cerebrum or cerebellum) is at its highest level. Accordingly, the highest brain pressure was reported at 389 ms after the start of the simulation for the deployed airbag and at 396 ms for the deactivated airbag—Figure 11 depicts the investigated time frames.

**Figure 12:**
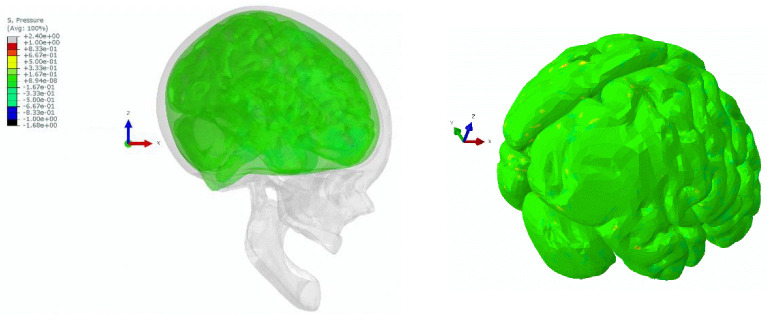
Contour plot of hydrostatic pressure (MPa) for the brain for the head–airbag impact at 389 ms.

The contour plot obtained in Abaqus CAE, depicted in Figure 12, revealed that the maximum hydrostatic pressure in the brain, for the case with deployed airbag, does not cause significant injuries. Some injury tolerance thresholds, which were put forward for the state-of-the-art finite element human model GHBMC (Global Human Body Models Consortium) indicate that the level of hydrodynamic pressure to cause a cerebral contusion shall be within the limits of −104 ÷ 237 kPa [16,43]. In the presented case, the overall pressure level is below 167 kPa (1.67 × 10^−1^ MPa) with some local, neglectable pick values generated by the finite element tetrahedral configuration—e.g., due to the local node-to-node contact interaction on the cortex. The coordinate system for the head remained unchanged, thus the head position is similar to the one presented in Figure 11.

**Figure 13:**
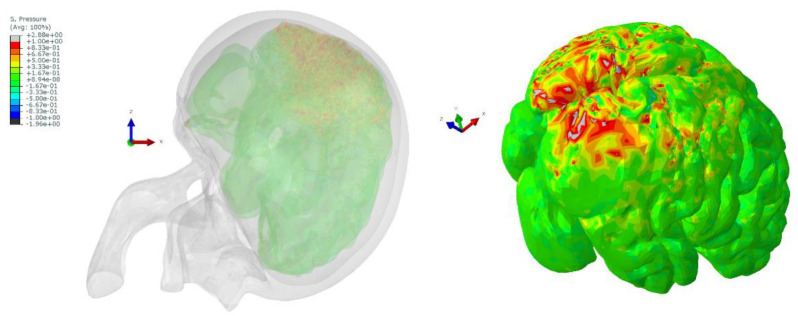
Contour plot of hydrostatic pressure (MPa) for the brain for the head–steering wheel impact at 396 ms—visible contrecoup phenomenon on the occipital region.

Unlike in the above case with the deployed airbag, we noticed that during the head–steering wheel impact, there is a major pressure gradient (>600 kPa) in the occipital region of the brain. Upon the head impact, the skull and the brain tend to move towards the impact site, creating an area of elevated pressure where the intracranial tissues are compressed and stretched. Based on the above criteria, the registered pressure would cause a major cerebral contusion. This is known as contrecoup injury. Figure 13 illustrates the pressure gradients across the brain, showing contrecoup phenomena during the simulation.

The present analysis includes some limitations. A rigid skull was used to predict the resulting brain injuries. However, considering the peak acceleration and HIC values achieved for the case without an airbag, this would probably result in a skull fracture. Nevertheless, since the results clearly show the significance of the airbag in mitigating brain injuries, one can conclude that the results with a deformable skull would withdraw the same conclusions. Therefore, although the quantitative analysis and the predicted outcome may suffer relevant changes by having a deformable skull, the presented qualitative results are solid. Another limitation of the current study is the fact that it includes one accident scenario and one dummy (50th percentile male) with one particular disability.

## 4. Conclusions

The human body is a complex, multi-layered structure consisting of various types of tissues, bones, and fluids governed by complex materials laws and interactions. The limitation of in vivo studies on human samples has made numerical modelling a vital tool for studying injury outcomes. The authors built the numerical models which included passive safety systems operating in a vehicle. Using this method, we simulated different cases of the frontal crash of a car driven by a person with disabilities: with and without the activated frontal airbag. We analysed the disabled person model without a left leg using a knob on the steering wheel. In this study, we used four stages to assess the disabled driver’s safety, i.e., frontal crash with a rigid wall, positioning the dummy under the gravity load, global analysis of the head-on collision and the detailed analysis of the head injuries using a YEAHM model. The presented research, based on a finite element approach, provided valuable data on the biomechanics of the human body. We concluded that the activated airbag for a person with disabilities reduces the effects of asymmetries in the positioning of the belts and body support points affecting injury. Moreover, all the biomechanical parameters, which were analysed on the dummy (global approach) in this manuscript, i.e., HIC, seat belt contact force and Neck Injury Criterion (Nij) support the use of the airbag. Only biomechanical parameters describing the longitudinal and vertical displacement of the body’s centre of gravity were higher, but they do not significantly affect injuries. In addition, the in-depth investigation of the human head hydrostatic pressure during the accident resulted in the conclusion that using a frontal airbag can significantly lower the possibility of cerebral contusion.

## Data Availability

Not applicable.
